# Labelling changes in response to a tax on sugar-sweetened beverages, United Kingdom of Great Britain and Northern Ireland

**DOI:** 10.2471/BLT.19.234542

**Published:** 2019-09-23

**Authors:** Kawther M Hashem, Feng J He, Graham A MacGregor

**Affiliations:** a Wolfson Institute of Preventive Medicine, Barts and The London School of Medicine & Dentistry, Queen Mary University of London, Charterhouse Square, London, EC1M 6BQ, England.

## Abstract

**Objective:**

To evaluate the changes in sugar and energy labelling of carbonated sugar-sweetened soft drinks after the implementation of a tax on sugar-sweetened drinks in the United Kingdom of Great Britain and Northern Ireland.

**Methods:**

We visited nine main supermarkets before (May 2014) and after (April 2018) the tax came into effect and obtained data from product packaging and nutrition information panels of carbonated sugar-sweetened soft drinks. We used the paired *t*-test to assess differences in sugar and energy content of the same products between 2014 and 2018.

**Findings:**

We obtained data from 166 products in 2014 and 464 products in 2018, of which 83 products were the same in both years. Large variations in stated sugar content were found between the different carbonated sugar-sweetened soft drinks in both 2014 and 2018 for all products and for the 83 products. The mean sugar content of the 83 products decreased by 42% between 2014 and 2018, from 9.1 g/100 mL (standard deviation, SD: 3.3) to 5.3 g/100 mL (SD: 3.5; *P* < 0.001). The mean energy content decreased by 40%, from 38 kcal/100 mL (SD: 13) in 2014 to 23 kcal/100 mL (SD: 15) in 2018 (*P* < 0.001).

**Conclusion:**

The significant decreases in the labelling of sugar and energy content of carbonated sugar-sweetened soft drinks after the levy came into effect suggest this tax has been effective. The sugar content of drinks still varied considerably in 2018, suggesting further reductions in sugar content of these drinks is possible. The levy thresholds should be reduced and the tax increased to drive further reformulation of soft drinks to reduce their sugar content.

## Résumé

**Objectif:**

Évaluer les changements dans l'étiquetage des boissons gazeuses sucrées non alcoolisées, sur le plan de la teneur en sucre et de la valeur énergétique, après l'instauration d'une taxe sur les boissons sucrées au Royaume-Uni de Grande-Bretagne et d'Irlande du Nord.

**Méthodes:**

Nous nous sommes rendus dans neuf grands supermarchés avant (mai 2014) et après (avril 2018) l'entrée en vigueur de la taxe et avons recueilli des données sur les emballages et les encadrés d'information nutritionnelle de boissons gazeuses sucrées non alcoolisées. Nous avons eu recours à un test t d'échantillons appariés pour évaluer les différences au niveau de la teneur en sucre et de la valeur énergétique des mêmes produits entre 2014 et 2018.

**Résultats:**

Nous avons obtenu des données sur 166 produits en 2014 et sur 464 produits en 2018, parmi lesquels 83 produits identiques sur ces deux années. S'agissant de la teneur en sucre indiquée, de gros écarts ont été observés entre les différentes boissons gazeuses sucrées non alcoolisées, aussi bien en 2014 qu'en 2018, pour l'ensemble des produits et pour les 83 produits semblables. La teneur en sucre moyenne des 83 produits a diminué de 42% entre 2014 et 2018, passant de 9,1 g/100 mL (écart type, ET: 3,3) à 5,3 g/100 mL (ET: 3,5; P < 0,001). La valeur énergétique moyenne a diminué de 40%, passant de 38 kcal/100 mL (ET: 13) en 2014 à 23 kcal/100 mL (ET: 15) en 2018 (P < 0,001).

**Conclusion:**

La diminution notable, sur les étiquettes, de la teneur en sucre et de la valeur énergétique des boissons gazeuses sucrées non alcoolisées après l'entrée en vigueur de la taxe laisse penser que celle-ci a été efficace. La teneur en sucre des boissons variait encore considérablement en 2018, ce qui laisse penser qu'il est possible de la réduire encore davantage. Les seuils d'application de la taxe devraient être abaissés et cette dernière augmentée afin d'encourager la reformulation des boissons non alcoolisées dans le but de réduire leur teneur en sucre.

## Resumen

**Objetivo:**

Evaluar los cambios en el etiquetado sobre el azúcar y la energía de las bebidas gaseosas endulzadas con azúcar después de la aplicación de un impuesto sobre las bebidas endulzadas con azúcar en el Reino Unido de Gran Bretaña e Irlanda del Norte.

**Métodos:**

Se visitaron nueve supermercados principales antes (mayo de 2014) y después (abril de 2018) de que el impuesto entrara en vigor y se obtuvieron datos de los envases de los productos y de los paneles de información nutricional de las bebidas gaseosas endulzadas con azúcar. Se utilizó la prueba t pareada para evaluar las diferencias en el contenido de azúcar y energía de los mismos productos entre 2014 y 2018.

**Resultados:**

Se obtuvieron datos de 166 productos de 2014 y 464 de 2018, de los cuales 83 productos fueron los mismos en ambos años. Se encontraron grandes variaciones en el contenido de azúcar declarado entre las diferentes bebidas gaseosas endulzadas con azúcar en 2014 y 2018 para todos los productos y para los 83 productos. El contenido promedio de azúcar de los 83 productos disminuyó en un 42 % entre 2014 y 2018, de 9,1 g/100 ml (desviación estándar, DE: 3,3) a 5,3 g/100 ml (DE: 3,5; *P* < 0,001). El contenido promedio de energía disminuyó en un 40 %, de 38 kcal/100 ml (SD: 13) en 2014 a 23 kcal/100 ml (SD: 15) en 2018 (*P* < 0,001).

**Conclusión:**

Las importantes reducciones en el etiquetado sobre el contenido de azúcar y de energía de las bebidas gaseosas endulzadas con azúcar después de que la tasa entrara en vigor sugieren que este impuesto ha sido efectivo. El contenido de azúcar en las bebidas todavía varía considerablemente en 2018, lo que sugiere que es posible reducir aún más el contenido de azúcar de dichas bebidas. Los umbrales de la tasa deben reducirse y el impuesto debe aumentarse para impulsar una nueva reformulación de las bebidas gaseosas a fin de reducir su contenido de azúcar.

## ملخص

**الغرض:**

تقييم التغييرات في السكر وملصقات الطاقة الخاصة بالمشروبات الغازية المحلاة بالسكر بعد تطبيق الضريبة على المشروبات المحلاة بالسكر في المملكة المتحدة لبريطانيا العظمى وأيرلندا الشمالية.

**الطريقة:**

قم بزيارة تسعة من محال السوبر ماركت الرئيسية قبل (مايو/أيار 2014) وبعد (أبريل/نيسان 2018) أن أصبحت الضريبة سارية المفعول، وحصلنا على بيانات من عبوات المنتجات، وقوائم معلومات التغذية، الخاصة بالمشروبات الغازية المحلاة بالسكر. قمنا بالاستعانة باختبار *t *-test المقترن لتقييم الاختلافات في محتوى السكر والطاقة لنفس المنتجات بين عامي 2014 و 2018.

**النتائج:**

لقد حصلنا على بيانات من 166 منتجاً في عام 2014، و464 منتجاً في عام 2018، منها 83 منتجاً كانت هي نفسها في كلتا السنتين. تم اكتشاف اختلافات كبيرة في محتوى السكر المُشار إليه بين مختلف المشروبات الغازية المحلاة بالسكر في كل من 2014 و2018 لكل المنتجات، ولـ 83 منتجاً. انخفض متوسط ​​محتوى السكر في 83 منتجاً بنسبة 42% بين عامي 2014 و2018، من 9.1 جم/100 مل (الانحراف المعياري: 3.3) إلى 5.3 جرام/100 مل (الانحراف المعياري: 3.5؛ *نسبة الاحتمال *أقل من **0.001). انخفض متوسط ​​محتوى الطاقة بنسبة 40%، من 38 كيلو كالوري/100 مل (الانحراف المعياري: 13) في عام 2014 إلى 23 كيلو كالوري إلى 5.3 جرام/100 مل (الانحراف المعياري: 15) في 2018 ( *نسبة الاحتمال *أقل من **0.001).

**الاستنتاج:**

تشير الانخفاضات الكبيرة في ملصقات محتوى السكر والطاقة في المشروبات الغازية المحلاة بالسكر بعد بدء سريان الضريبة إلى أن هذه الضريبة كانت فعالة. لا يزال محتوى السكر في المشروبات متنوعاً بشكل كبير في عام 2018، مما يشير إلى أن إجراء المزيد من التخفيضات في محتوى السكر بهذه المشروبات أمراً ممكناً. يجب تقليل حدود الضريبة وزيادة الضريبة، لتحفيز المزيد من إعادة الصياغة للمشروبات الغازية بهدف تقليل محتواها من السكر. ****

## 摘要

**目的:**

旨在评估大不列颠及北爱尔兰联合王国对含糖饮料推行征税后，含糖碳酸软饮料产品标签上糖份和能量数据的变化。

**方法:**

我们在该税收政策推行前（2014 年 5 月）与正式生效后（2018 年 4 月）分别走访了 9 个大型超市，并从含糖碳酸软饮料的产品包装及其营养信息表中获取了数据。我们使用配对 *t* 检验评估了 2014 年和 2018 年相同产品中糖份和能量含量的差异。

**结果:**

我们从 2014 年售卖的 166 种产品和 2018 年售卖的 464 种产品中获取数据，其中完全相同的产品有 83 种。。2014 年和 2018 年的数据均显示，包括这 83 种相同产品在内的所有不同含糖碳酸软饮料产品之间的含糖量差异很大。2014 年至 2018 年期间，这 83 种产品的平均含糖量下降了 42％，从 9.1 克/100 毫升（标准差：3.3）降至 5.3 克/100 毫升（标准差：3.5；P < 0.001）。平均能量含量下降了 40%，从 2014 年的 38 千卡/100 毫升（标准差：13）降至 2018 年的 23 千卡/100 毫升（标准差：15） (*P* < 0.001)。

**结论:**

该税收政策正式生效后，含糖碳酸软饮料标签上显示的糖份和能量含量显著降低，这一税收政策是有效的。2018 年，各种含糖饮料之间的含糖量仍然相差很大，这表明这些饮料的含糖量仍有继续下降的可能。应降低税收起征点，并增加税收，以推动行业内针对软饮料的进一步配方调整，从而降低含糖量。****

## Резюме

**Цель:**

Оценка изменений в информации о содержании сахара и энергетической ценности подслащенных безалкогольных напитков после ввода налога на подслащенные напитки в Соединенном Королевстве Великобритании и Северной Ирландии.

**Методы:**

Авторы посетили девять крупных супермаркетов до введения налога (в мае 2014 г.) и после его вступления в силу (в апреле 2018 г.) и получили данные о том, какая информация содержится на упаковке продукции и на участках этикеток, отведенных под информацию о питательной ценности газированных подслащенных безалкогольных напитков. Авторы использовали парный *t*-критерий для определения разницы в указании содержания сахара и энергетической ценности одних и тех же продуктов в период между 2014 и 2018 годами.

**Результаты:**

Были получены данные по 166 продуктам в 2014 году и 464 продуктам в 2018 году, из которых 83 продукта совпадали. Значительные вариации в указании содержания сахара были обнаружены у различных газированных подслащенных безалкогольных напитков и в 2014 году, и в 2018 году как в целом, так и для 83 одинаковых продуктов. Среднее содержание сахара в 83 продуктах упало за период с 2014 по 2018 год на 42%: с 9,1 г/100 мл (среднеквадратичное отклонение, СО: 3,3) до 5,3 г/100 мл (СО: 3,5; *P* < 0,001). Средняя энергетическая ценность снизилась на 40%: с 38 ккал/100 мл (СО: 13) в 2014 году до 23 ккал/100 мл (СО: 15) в 2018 году (*P* < 0,001).

**Вывод:**

Значительное снижение содержания сахара и энергетической ценности газированных подслащенных безалкогольных напитков после ввода налога на сахар, прослеживаемое по данным на этикетках товаров, позволяет предположить, что введение налога оказалось эффективным. В 2018 году содержание сахара в напитках сильно варьировалось, что позволяет предположить, что возможно дальнейшее снижение уровня сахара в этих напитках. Необходимо снизить пороги налогообложения и увеличить налог, чтобы содействовать дальнейшему изменению состава напитков для снижения содержания сахара.****

## Introduction

In July 2015, the Scientific Advisory Committee on Nutrition of the United Kingdom of Great Britain and Northern Ireland recommended that average free sugars (sugar) intake across the country’s population should not exceed 5% of total energy intake.^1^ The committee defined free sugars as all monosaccharides and disaccharides added to foods by the manufacturer, cook or consumer, as well as sugars naturally present in honey, syrups (e.g. high fructose corn syrup, glucose syrup and maple syrup) and unsweetened fruit juices. Free sugars do not include lactose when naturally present in milk and milk products, nor sugars contained within the cellular structure of foods (whole fruits and vegetables).^1^ However, this definition is new and not aligned with current nutrition labelling on food packaging and manufacturers’ claims about sugar content. Manufacturers can still claim that their product contains “no added sugar” even though the product contains fruit or vegetable juice or juice concentrate.

The committee on nutrition also advised that consumption of sugar-sweetened drinks be minimized in children and adults,^1^ because high intake of sugar is contributing to obesity, type 2 diabetes and dental caries,^2^^–^^10^ all of which are major public health problems in the United Kingdom.^11^^–^^18^

The average sugar intake in 2014 in the United Kingdom exceeded the recommendations of the committee on nutrition in all age groups.^19^ The mean sugar intake in adults was 60 g per day, accounting for 12% of daily energy intake. The mean sugar intake in children was 54 g (13.4% of energy intake) per day in 4–10-year-olds and 73 g (14.1%) per day in 11–18-year-olds.^19^

Soft drinks, which include all drinks and fruit juices apart from alcoholic drinks, are the main contributors to sugar intake in children (4–10 years) and teenagers (11–18 years), and the second main contributor in adults (18–64 years).^20^ Within soft drinks, carbonated soft drinks were the single largest category of the soft drinks market in 2016 with a 38% market share by volume.^21^ The volume of carbonated soft drinks sold was 5 billion litres, with an average person consuming 78 L of carbonated soft drinks per year, of which 50% were high sugar drinks.^22^

In 2016, the British government announced a tax on sugar-sweetened drinks, the Soft Drinks Industry Levy, to tackle childhood obesity. This tax came into effect in April 2018. The drinks liable for this tax are those that have had sugar added during production, including honey or syrups. Drinks are not liable if they have fruit juice, vegetable juice and milk in them unless these are in addition to sugar.^23^ Companies producing sugar-sweetened soft drinks will have to pay 0.24 British pounds (£) (equivalent to 0.32 United States dollars, US$, in 2018) per litre of drink if the drink contains 8 g or more of sugar per 100 mL or £0.18 (US$ 0.24) per litre of drink if it contains between 5 and 8 g of sugar per 100 mL. Companies can reformulate their soft drink to reduce the sugar content, which may reduce or remove the tax liability.

Product reformulation is commonly described as a voluntary or mandatory effort by government to get manufacturers to lower the unhealthy components (e.g. saturated fat, trans fats, sugar, salt) of food and drink products at the time of production, without making the profile of the overall product worse for consumers (e.g. increasing calorie content).^24^ The reformulated products often replace an existing product (e.g. the same brand of drink with less sugar). The British government aims to motivate manufacturers of sugar-sweetened soft drinks with 8 g of sugar per 100 mL or more to reduce the levels to below 8 g and pay a lower tax, and manufacturers of drinks with 5–8 g of sugar per 100 mL to lower the sugar levels to less than 5 g and pay no tax.^23^

This study aimed to assess changes in the sugar and energy content on labels of carbonated sugar-sweetened soft drinks between 2014 and 2018 after the implementation of the tax on sugar-sweetened drinks in United Kingdom.^23^ The 2014 data, which were previously published,^25^ found that the sugar content of similar products and flavours varied substantially, which suggests that products with lower sugar content were available and that those products with higher content could be reformulated to reduce the amount of sugar.

## Methods

### Study design and setting

The same study design and procedures were used in both 2014 and 2018.^25^ We assessed all carbonated sugar-sweetened soft drinks available in the nine main supermarkets in the United Kingdom in May 2014 and April 2018. The retailers were Aldi, Asda, Lidl, Marks and Spencer, Morrisons, Sainsbury’s, Tesco, Co-op^®^ Food and Waitrose, which together have over 93.2% of the grocery market share in the country.^26^

We checked the products in one large outlet of each of the retailers because the bigger stores tend to stock more own-label and branded products than their smaller branches. Data were collected from the product packaging and nutrient information panels on the products.

### Data collection

We defined carbonated sugar-sweetened soft drinks as any carbonated or sparkling drink with added sugar that are not described as energy or sport’s drinks on the pack. 

Since we focused on the sugar and energy content of drinks, we excluded carbonated sugar-sweetened soft drinks in 2014 labelled as zero or no added sugar. However, in 2018, we included all carbonated sugar-sweetened soft drinks, regardless of their claims. We included all drinks because some manufacturers were claiming that their products contained no added sugar, even when they contained fruit juice or juice concentrates.

We manually collected nutrition information from product packaging and nutrition labelling using the data collector applications of Foodswitch United Kingdom, which can be used to take images of food packaging for manual data entry on a database, from which the data can be extracted for analysis.^27^ For each product, we collected data on the name of the company that manufactured the product (e.g. The Coca-Cola^®^ Company), brand name of the soft drink (e.g. Fanta™), product name (e.g. Fanta™ Orange, Fanta™ Lemon), pack size, serving size, amounts of sugar (g) and energy (kcal) in the product, per 100 mL and per serving.

We checked all the data again after entry and 5% of the entries against the product label in a random selection of products. We also screened each flavour category for outliers and checked the lowest and highest values in each category against the product labels and corrected, if necessary.

We divided the products into categories of flavour (Box 1), supermarket own label and brand, and manufacturer. If there were more than four products with a particular flavour, we created a category. Products that did not fit into a flavour category were included in the all-products analysis.

Box 1 **Categories of flavour of sugar-sweetened carbonated soft drinks**

**Carbonated drinks flavoured with:**

• apple

• cherry

• elderflower

• grape

• orange

**Carbonated drinks described as:**

• cola

• cream soda and also flavoured (e.g. cream soda with raspberry)

• dandelion and burdock

• flavoured cola (e.g. cola with cherry or vanilla)

• ginger ale

• ginger beer, or root beer or ginger brew

• iron brew or similar

• lemonade

• tonic water

Note: If there were more than four products with a particular flavour, we created a category.

### Analysis

#### Per 100 mL

Some brands sell the same formulation in different serving sizes, e.g. Coca-Cola^®^ comes in 250 mL and 330 mL cans and 500 mL bottles. The formulation per 100 mL is the same. Therefore, for the data per 100 mL, we only included an example of one formulation regardless of the serving size.

#### Per serving

The data per serving included all the different serving sizes available except for 1 L bottles. We excluded 1 L products from the per serving analysis since we could not accurately quantify how much a consumer would drink if they were drinking from a 1 L bottle, but they are likely to drink more than the industry-standard serving of 250 mL. We considered any product with a can or bottle size up to 500 mL as one serving, regardless of what is stated on the product as a serving size. For example, often a bottle of 500 mL is split into two servings, but we consider that most consumers drink these drinks as one serving.

#### Level of sugar

We compared the sugar content of the products to the United Kingdom front-of-pack colour-coded labelling for sugar in drinks: red/high sugar > 13.5 g/portion or > 11.25 g/100 mL; amber/medium sugar > 2.5 to ≤ 11.25 g/100 mL; and green/low sugar ≤ 2.5 g/100 mL.^28^ The criteria for portion size applied to portion/serving sizes more than 150 mL.

#### Maximum sugar intake

We also compared the sugar content of the products with the maximum daily recommendation for sugar intake for adults (30 g) and 7–10-year-old children (24 g).^1^

#### Soft Drinks Industry Levy

We assessed the products against the levy thresholds: £0.24 per litre of drink if the drink contains 8 g of sugar or more per 100 mL or £0.18 per litre if the drink contains between 5 and 8 g of sugar per 100 mL.^23^

#### Manufacturers

We analysed the sugar and energy content by manufacturer for each manufacturer with five or more products in the sample in both 2014 and 2018. Where a product was imported, but produced by a recognized international manufacturer, we categorized it under the international manufacturer, e.g. a product produced by Coca-Cola**^®^** New Zealand was categorized under Coca-Cola**^®^**. This categorization helps show the contribution of each manufacturer’s products to sugar content in soft drinks and helps in tracking companies’ reformulation progress over time.

### Statistical analysis

We used the independent-sample *t*-test to compare the levels of sugar between supermarket own-label and brand products in the entire samples of products in 2014 and 2018. We assumed that supermarkets are more willing to reformulate their own-label products than brand manufacturers who may be more risk averse and reluctant to reformulate products. This reluctance may be because brand manufacturers are influenced by loyalty of their customers to the brand and so they want to continue using old recipes that customers are used to, while supermarkets are constantly changing products and ranges and so are more willing to take risks.

We used the paired *t*-test to examine whether the sugar content of drinks changed significantly between 2014 and 2018. For assessing reformulation, we only included products with data available in both 2014 and 2018 in this analysis.

Descriptive statistics (mean, standard deviation, SD, and range) were calculated. *P*-values of less than 0.05 were considered statistically significant. All data were analysed using SPSS, version 25 (SPSS Inc., Chicago, United States of America).

## Results

**Fig. 1:**
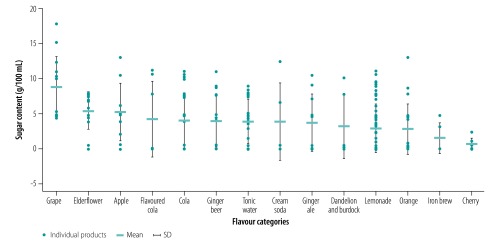
Sugar content in different flavour categories of carbonated soft drinks in the United Kingdom of Great Britain and Northern Ireland, 2018

We collected 166 and 464 products with nutrition information in 2014 and 2018, respectively. Of these products, 166 and 381 carbonated sugar-sweetened soft drinks met the inclusion criteria per 100 mL in 2014 and 2018, respectively. In 2018, the sample was larger because we included products that claimed to have zero sugar and no added sugar. The mean sugar content per 100 mL was 9.1 g (SD: 3.2) in 2014 and 4.4 g (SD: 4.0) in 2018, with large variations in sugar content between different drinks ranging from 1.0 to 16.0 g in 2014 and 0.0 to 17.9 g in 2018 (Table 1 and Fig. 1). In an analysis of the 249 products without claims of zero sugar and no added sugar in 2018, the mean sugar content was still lower than in 2014, 6.4 g/100 mL (SD: 3.4).

**Table 1:** Sugar content according to label of flavour categories of carbonated soft drinks, United Kingdom of Great Britain and Northern Ireland, 2014 and 2018

Category^a^	2014				2018^b^		
	No. of drinks	Sugar content g/100 mL			No. of drinks	Sugar content g/100 mL	
		Mean (SD)	Range			Mean (SD)	Range
Grape	4	12.4 (1.0)	11.5–13.5		15	8.8 (4.3)	4.3–17.9
Elderflower	7	7.6 (1.5)	4.9–10.0		13	5.5 (2.7)	0.0–8.0
Apple	3	6.8 (4.2)	2.1–10.2		10	5.2 (4.1)	0.0–13.0
Flavoured cola	3	11.1 (0.5)	10.6–11.6		7	4.2 (5.4)	0.0–11.2
Cola	16	10.7 (0.5)	9.7–12.1		31	4.1 (4.0)	0.0–11.0
Ginger beer	18	11.8 (3.0)	5.6–16.0		18	4.0 (3.7)	0.0–11.0
Tonic water^c^	–	–	–		36	3.9 (3.2)	0.0–8.9
Cream soda	4	9.8 (2.5)	6.1–11.5		5	3.9 (5.5)	0.0–12.4
Ginger ale	11	6.9 (2.3)	3.8–9.2		10	3.7 (4.1)	0.0–10.5
Dandelion and burdock	4	8.2 (3.3)	4.2–11.8		6	3.2 (4.5)	0.0–10.1
Lemonade	38	7.4 (3.5)	1.0–13.5		65	3.0 (3.5)	0.0–11.1
Orange	11	9.8 (3.3)	1.9–14.3		20	2.8 (3.6)	0.0–13.0
Iron brew	2	7.3 (4.6)	4.0–10.5		5	1.6 (2.2)	0.0–4.7
Cherry	2	5.6 (4.5)	2.4–8.8		7	0.7 (0.8)	0.0–2.4
Other^d^	43	NA	NA		133	NA	NA
**All products**	**166**	**9.1 (3.2)**	**1.0–16.0**		**381**	**4.4 (4.0)**	**0.0–17.9**

SD: standard deviation; NA: not applicable.

^a^ In descending order of sugar content by 2018 data.

^b^ Includes products claiming to have zero sugar or no added sugar.

^c^ Was not included in the 2014 study.

^d^ These products did not fit into any flavour category and were not analysed separately, but were included in the all-products analysis.

SD: standard deviations.

In 2014, on average, grape drinks contained the highest amount of sugar, ranging from 11.5 to 13.5 g/100 mL, followed by ginger beer (5.6 to 16.0 g/100 mL). Cherry-flavoured drinks contained the lowest amount of sugar (Table 1). Similarly, in 2018, on average, grape drinks contained the highest amount of sugar, ranging from 4.3 to 17.9 g/100 mL, followed by elderflower-flavoured drinks. 

Table 2 shows the energy content in different categories of carbonated drinks per 100 mL in 2014 and 2018. The mean energy content per 100 mL was 38 kcal (SD: 13) in 2014 and 19 kcal (SD: 16) in 2018, with large variations in energy content between different drinks, ranging from 5 to 65 kcal in 2014 and 0 to 72 kcal in 2018. Grape drinks contained the highest amount of energy on average in both 2014 and 2018.

**Table 2:** Energy content according to label of flavour categories of carbonated soft drinks, United Kingdom of Great Britain and Northern Ireland, 2014 and 2018

Category^a^	2014				2018^b^		
	No. of drinks	Energy content kcal/100 mL			No. of drinks	Energy content kcal/100 mL	
		Mean (SD)	Range			Mean (SD)	Range
Grape	4	52 (3)	49–56		15	38 (17)	20–72
Elderflower	3	30 (19)	10–47		10	23 (17)	1–53
Apple	7	31 (5)	24–40		13	23 (11)	1–32
Flavoured cola	3	45 (2)	43–47		7	18 (22)	0–45
Cola	–	–	–		36	18 (13)	1–37
Ginger beer	18	49 (12)	25–65		18	17 (16)	0–46
Tonic water^c^	11	29 (9)	17–38		10	17 (17)	1–45
Cream soda	16	44 (2)	39–49		31	16 (16)	0–43
Ginger ale	4	40 (10)	26–47		5	16 (22)	1–50
Dandelion and burdock	4	34 (11)	21–47		6	16 (22)	1–47
Lemonade	38	32 (14)	5–57		65	14 (15)	0–48
Orange	11	41 (13)	10–58		20	13 (15)	1–54
Iron brew	2	30 (18)	17–43		5	7 (9)	1–20
Cherry	2	23 (17)	11–35		7	4 (4)	1–11
Other^d^	43	NA	NA		133	NA	NA
**All products**	**166**	**38 (13)**	**5–65**		**381**	**19 (16)**	**0–72**

SD: standard deviation; NA: not applicable.

^a^ In descending order of energy content by 2018 data.

^b^ Includes products claiming to have zero sugar or no added sugar.

^c^ Was not included in the 2014 survey.

^d^ These products did not fit into any flavour category and were not analysed separately, but were included in the all-products analysis.

When we excluded the zero and no added sugar products from the 2018 data, the mean energy content in 2018 was still lower than 2014 at 27 kcal/100 mL (SD: 14).

### Manufacturer comparison

As shown in Table 3, there was a significant difference in sugar content between supermarket own-label and brand products in 2014 (8.4 g vs 9.6 g/100 mL, *P* = 0.023) and 2018 (3.0 g vs 5.8 g/100 mL, *P* < 0.001). 

**Table 3:** Sugar content of carbonated soft drinks according to label, by manufacturer, United Kingdom of Great Britain and Northern Ireland, 2014 and 2018

Manufacturer name	2014				2018			Mean difference g/100 mL
	No. of drinks	Sugar content g/100 mL			No. of drinks	Sugar content g/100 mL		
		Mean (SD)	Range			Mean (SD)	Range	
**Combined manufacturers**								
Supermarket own-labels	68	8.4 (3.1)^a^	1.0–13.9		187	3.0 (3.8)^b^	0.0–17.9	−5.4
Brands	98	9.6 (3.2)^a^	1.9–16.0		194	5.8 (3.6)^b^	0.0–15.1	−3.8
**Individual manufacturer^c^**								
Tropical Sun	1	13.0 (0.0)	13.0–13.0		5	12.3 (3.8)	7.6–15.1	−0.7
Twiss™	–	–	–		5	9.1 (2.3)	6.9–12.0	NA
Franklin & Sons Ltd	–	–	–		6	8.7 (2.4)	4.3–11.0	NA
S.Pellegrino®	5	10.9 (1.1)	9.7–12.1		5	8.6 (2.3)	4.7–10.3	−2.3
Merrydown	6	11.3 (0.4)	10.7–11.6		9	7.7 (2.4)	5.3–10.9	−3.6
Fentimans	8	8.9 (3.2)	4.9–11.8		9	7.6 (0.3)	6.9–7.8	−1.3
Belvoir Fruit Farms	3	9.6 (1.5)	8.0–10.9		13	6.4 (2.1)	4.1–12.0	−3.2
Fever-tree	2	9.5 (0.7)	9.0–10.0		12	6.1 (2.1)	2.9–8.4	−3.4
PepsiCo	1	10.6 (0.0)	10.6–10.6		4	6.0 (7.0)	0.0–13.0	−4.6
Bottlegreen Drinks Co.	5	7.1 (0.1)	7.0–7.3		8	5.8 (1.3)	3.9–7.0	−1.3
Marks & Spencer	12	9.2 (2.5)	4.9–13.8		38	5.7 (5.2)	0.0–17.9	−3.5
Cott Beverages Ltd	7	13.2 (2.5)	8.4–16.0		9	5.5 (4.7)	0.0–13.5	−7.7
Britvic® plc	16	6.7 (4.4)	1.9–14.3		15	4.5 (2.9)	0.0–13.0	−2.2
The Coca-Cola® Company	20	9.2 (2.0)	4.2–13.5		34	4.4 (4.1)	0.0–12.3	−4.8
AG Barr	5	9.7 (2.3)	5.7–11.5		18	3.6 (1.9)	0.0–4.9	−6.1
Waitrose	10	10.3 (2.0)	7.3–13.9		16	3.3 (4.0)	0.0–9.9	−7.0
Lidl	2	10.5 (0.6)	10.0–10.9		14	3.0 (4.0)	0.0–10.3	−7.5
Sainsbury's	10	10.7 (2.1)	8.1–13.7		27	2.8 (3.1)	0.5–10.6	−7.9
Co-op® Food	6	5.8 (3.4)	1.0–10.4		12	2.8 (3.6)	0.0–11.0	−3.0
ASDA	10	6.2 (2.4)	3.5–10.7		13	2.2 (2.4)	0.0–7.4	−4.0
Morrisons	7	5.8 (3.4)	3.6–10.9		25	1.7 (3.1)	0.0–11.1	−4.1
Aldi	2	7.6 (4.3)	4.5–10.6		13	1.6 (1.8)	0.5–4.9	−6.0
Tesco	9	9.0 (2.9)	4.1–13.3		29	1.4 (2.0)	0.0–4.9	−7.6
Princes Ltd	6	13.3 (1.2)	12.1–15.6		–	–	–	NA
Other	13	NA	NA		42	NA	NA	NA
**All products**	**166**	**9.1 (3.2)**	**1.0–16.0**		**381**	**4.4 (4.0)**	**0.0–17.9**	**−4.7**

SD: standard deviation; NA: not applicable.

^a^ The difference in mean sugar content between own label and brand products is statistically significant (P = 0.023).

^b^ The difference in mean sugar content between own label and brand products is statistically significant (P < 0.001).

^c^ In descending order of sugar content by 2018 data.

Note: Dashes indicate that the products from these manufacturers were not found in the stores in 2014 or 2018.

In 2014, the Princes Ltd product range contained the highest average sugar content per 100 mL (13.3 g; SD: 1.2) and the Co-op^®^ Food range contained the lowest (5.8 g; SD: 3.4; Table 3). In 2018, the Tropical Sun range contained the highest average sugar content per 100 mL (12.3 g; SD: 3.8) and Tesco had the lowest (1.4 g; SD: 2.0). In all the products, Sainsbury’s made the biggest reduction in sugar content on average between 2014 and 2018, a reduction of 7.9 g/100 mL.

In 2018, the Tropical Sun product range contained the highest energy content per 100 mL and Tesco and Aldi the lowest (Table 4). In all the samples Cott Beverages Ltd and Sainsbury’s made the biggest reduction in energy content on average between 2014 and 2018, a reduction of 32 kcal/100 mL.

**Table 4:** Energy content in carbonated soft drinks according to label, by manufacturer, United Kingdom of Great Britain and Northern Ireland, 2014 and 2018

Manufacturer name	2014				2018			Mean difference g/100 mL
	No. of drinks	Energy content kcal/100 mL			No. of drinks	Energy content kcal/100 mL		
		Mean (SD)	Range			Mean (SD)	Range	
**Combined manufacturers**								
Supermarket own-labels	68	36 (13)	5–65		187	13 (16)	0–72	−23
Brands	98	40 (13)	9–64		194	25 (15)	0–57	−15
**Individual manufacturer^a^**								
Tropical Sun	1	51 (0)	51–51		5	47 (13)	32–57	–4
S.Pellegrino®	5	48 (4)	43–54		5	37 (10)	20–42	−11
Fentimans	8	36 (12)	21–47		9	37 (5)	29–47	1
Franklin & Sons	–	–	–		6	36 (9)	19–46	NA
Twiss™	–	–	–		5	36 (7)	29–45	NA
Merrydown	6	47 (2)	45–49		9	34 (10)	23–45	−13
Belvoir Fruit Farms	3	39 (6)	32–44		13	27 (9)	20–52	−12
Fever-tree	2	41 (4)	39–44		12	27 (11)	7–36	−14
Bottlegreen Drinks Co.	5	30 (0)	30–30		8	25 (5)	20–30	−5
Marks & Spencer	12	39 (11)	24–65		38	25 (21)	0–72	−14
Cott Beverages Ltd	7	55 (9)	35–64		9	23 (18)	1–54	−32
PepsiCo	1	43 (0)	43–43		4	22 (26)	0–48	−21
Britvic® plc	16	28 (17)	9–58		15	20 (12)	2–54	−8
The Coca-Cola® Company	20	39 (8)	19–56		34	19 (17)	0–53	−20
AG Barr	5	40 (9)	23–47		18	16 (8)	1–22	−24
Waitrose	10	42 (9)	29–57		16	15 (16)	0–40	−27
Lidl	2	43 (3)	42–45		14	14 (18)	1–49	−29
Sainsbury's	10	45 (9)	33–57		27	13 (14)	1–44	−32
Co-op® Food	6	23 (14)	5–43		12	12 (14)	1–42	−11
ASDA	10	27 (10)	15–44		13	10 (11)	0–30	−17
Morrisons	7	26 (14)	15–48		25	8 (13)	1–48	−18
Aldi	2	32 (16)	20–43		13	7 (10)	0–28	−25
Tesco	9	37 (11)	18–55		29	7 (8)	1–21	−30
Princes Ltd	6	54 (5)	49–64		–	–		NA
Other	13	NA	NA		42	NA	NA	NA
**All products**	**166**	**38 (13)**	**5–65**		**381**	**19 (16)**	**0–72**	**−19**

SD: standard deviation; NA: not applicable.

^a^ In descending order of energy content by 2018 data.

Note: Dashes indicate that the products from these manufacturers were not found in the stores in 2014 or 2018.

### Sugar content per serving

We included 43 and 169 products in the analysis per serving in 2014 and 2018, respectively. The serving size ranged from 250 to 500 mL in 2014 and from 150 to 500 mL in 2018. In 2018, 18% (31/169) of drinks were sold in a 500 mL bottle, 44% (74/169) in a 330 mL can, 22% (38/169) in 250 mL and 5% (9/169) in a 150 mL can or bottle. Other products came in less standard sizes: 11 were in 275 mL cans or bottles, four in 200 mL and one each in 420 mL, 400 mL and 350 mL.

In 2014, 70% (30/43) of the products and in 2018, 17% (29/169) of the products exceeded the maximum daily recommendation for sugar intake per serving for an adult (30 g). In addition, 84% (36/43) in 2014 and 27% (45/169) in 2018 of the products exceeded the maximum daily recommendation for sugar intake for a child aged 7–10 years (24 g).

### Changes between 2014 and 2018

#### Reformulation

We included the same 83 products in both 2014 and 2018. The mean sugar content per 100 mL for these products was 9.1 g (SD: 3.3) in 2014 and 5.3 g (SD: 3.5) in 2018, a reduction in sugar content of 42% (*P* < 0.001 by paired *t*-test). The mean energy content per 100 mL was 38 kcal (SD: 13) in 2014 and 23 kcal (SD: 15) in 2018, a 40% reduction in energy content (*P* < 0.001 by paired *t*-test). These averages are slightly different from those when all products were included in each year. This analysis reflects reductions made in the same products rather than the overall products available.

Of the 83 drinks included in both years, 23% (19/83) had a red (high) label for sugar (> 11.25 g/100 mL), 71% (59/83) amber and 6% (5/83) green in 2014. In 2018, only 1% (1/83) had a red label, 72% (60/83) amber and 27% (22/83) green. However, since most of the drinks were sold in serving sizes bigger than 150 mL, 38% (26/69) of drinks in 2018 had a red label for per serving (> 13.5 g/portion), applied only to products sold as a single serving up to 500 mL (1 L bottles were excluded from this analysis).

#### Soft Drink Industry Levy

In terms of the levy thresholds,^23^ 71% (118/166) of the products in 2014 contained 8 g/100 mL or more sugar, 13% (22/166) contained < 8 to > 5 g/100 mL and only 16% (26/166) had 5 g/100 mL or less sugar. By 2018, 18% (69/381) of the products contained 8 g/100 mL or more sugar, 19% (73/381) contained < 8 to > 5 g/100 mL and 63% (239/381) were below the 5 g/100 mL sugar threshold.

## Discussion

Our study shows early changes in the sugar content on labels of carbonated sugar-sweetened soft drinks in the United Kingdom. The comparisons made between the same products over time showed significant reductions in the sugar content of these drinks. These results illustrate the substantial reformulation manufacturers have made so far. The reductions seen are likely to have been prompted by the Soft Drink Industry Levy.^23^ Before the announcement of this tax, only Tesco and ASDA had publicly announced their intention to gradually reformulate their products to reduce the sugar content but the tax seems to have speeded up the process.^29^^–^^31^ After the announcement of the Soft Drink Industry Levy with the two-year window given for reformulation, many companies made public announcements about reformulating their products to avoid the tax.^31^^–^^35^ These announcements support our belief that most of the reductions in sugar seen in our study can be attributed to the Soft Drink Industry Levy.

Since the implementation of the Soft Drink Industry Levy, sales of carbonated sugar-sweetened soft drinks have also fallen, resulting in a decrease in overall consumption of these drinks,^36^ as was speculated.^37^ Other countries have imposed taxes on sugar-sweetened drinks and some have also seen reductions in sales.^38^^,^^39^

Despite this reduction, sugar content remains high: many of the drinks in 2018 would still receive a red (high) label for sugars per serving. The sugar content of similar products varied considerably, judging by the wide ranges, which demonstrates that further reformulations are possible. If further reductions are needed, and if they are based on the 2018 data on sugar and energy content, the thresholds of the Soft Drink Industry Levy could be reduced and the tax paid increased to drive further incremental reformulation.

We also analysed the sugar content of soft drinks by manufacturer, which showed that the products that were high in sugar were generally produced by a few main brand manufacturers. These manufacturers tend just to pay the tax instead of reformulating their products to reduce sugar content. Reformulation is the responsibility of the manufacturers and they need to be targeted with measures that push them to reformulate their products, such as higher taxes and/or lower thresholds.

Manufacturers of the reformulated products in our study have either reduced the total sugar or reduced sugar by replacing it with non-caloric sweeteners without changing the product name. Replacing sugar with non-caloric sweeteners has been associated with a lower risk of obesity.^40^ Nevertheless, there is insufficient evidence on the relationship between the use of non-caloric sweeteners and long-term weight control.^41^

Future research can focus on continuing the collation and documentation of sugar and energy content of products. Our study provides categories of products that can be monitored in the future. The data collected can be combined with sales data of best-selling products to monitor change over time and the effect on the intake of sugar in the population. 

Our study has limitations. We used sugar and energy content data provided on the nutrition labels of drinks products that were available in stores on the days the products were collected. Therefore, our data are dependent on the accuracy of the data provided on the label and the availability of products in stores. Manufacturers are assumed to provide accurate and up-to-date information on their packaging in line with European Union regulations. Moreover, most products are required by law to have nutrition information on packaging in the United Kingdom and manufacturers will likely provide the correct figures. Therefore, the data collected from the label are likely to match the true sugar content.

Products available on supermarket shelves are not always strictly in line with the descriptions used by the industry. For instance, carbonated sugar-sweetened soft drinks in 2014 labelled as zero sugar or no added sugar were excluded. However, in 2018, we included all these drinks, regardless of such claims. We decided to include these drinks because some manufacturers claimed their products contained no added sugar, but they still added juice or juice concentrates to them which contain sugar. To minimize the potential influence of the products claiming zero sugar and no added sugar in 2018, we did separate analyses that (i) included only the products surveyed in both 2014 and 2018 and (ii) excluded products claiming zero and no added sugar in both years. These analyses showed a consistent and significant reduction in sugar in carbonated sugar-sweetened soft drinks.

Reformulations to reduce sugar in carbonated soft drinks are contributing to achieving the recommendation that sugar intake should not exceed 5% of total energy intake.^1^

## References

[1] Why 5%? London: Public Health England; 2015. Available from: https://www.gov.uk/government/uploads/system/uploads/attachment_data/file/446010/Why_5__-_The_Science_Behind_SACN.pdf [cited 2017 Jul 1].

[2] Scientific Advisory Committee on Nutrition. Carbohydrates and health. London: TSO; 2015. Available from: https://www.gov.uk/government/uploads/system/uploads/attachment_data/file/445503/SACN_Carbohydrates_and_Health.pdf [cited 2018 Mar 2].

[3] Romaguera D, Norat T, Wark PA, Vergnaud AC, Schulze MB, van Woudenbergh GJ, et al. InterAct Consortium. Consumption of sweet beverages and type 2 diabetes incidence in European adults: results from EPIC-InterAct. Diabetologia. 2013 7;56(7):1520–30. 10.1007/s00125-013-2899-8 23620057

[4] de Koning L, Malik VS, Rimm EB, Willett WC, Hu FB. Sugar-sweetened and artificially sweetened beverage consumption and risk of type 2 diabetes in men. Am J Clin Nutr. 2011 6;93(6):1321–7. 10.3945/ajcn.110.007922 21430119 PMC3095502

[5] Maki KC, Phillips AK. Dietary substitutions for refined carbohydrate that show promise for reducing risk of type 2 diabetes in men and women. J Nutr. 2015 1;145(1):159S–63S. 10.3945/jn.114.195149 25527674

[6] Feinman RD, Pogozelski WK, Astrup A, Bernstein RK, Fine EJ, Westman EC, et al. Dietary carbohydrate restriction as the first approach in diabetes management: critical review and evidence base. Nutrition. 2015 1;31(1):1–13. 10.1016/j.nut.2014.06.011 25287761

[7] Te Morenga L, Mallard S, Mann J. Dietary sugars and body weight: systematic review and meta-analyses of randomised controlled trials and cohort studies. BMJ. 2012 1 15;346:e7492. 10.1136/bmj.e7492 23321486

[8] Johnson RK, Appel LJ, Brands M, Howard BV, Lefevre M, Lustig RH, et al.; American Heart Association Nutrition Committee of the Council on Nutrition, Physical Activity, and Metabolism and the Council on Epidemiology and Prevention. Dietary sugars intake and cardiovascular health: a scientific statement from the American Heart Association. Circulation. 2009 9 15;120(11):1011–20. 10.1161/CIRCULATIONAHA.109.192627 19704096

[9] Xi B, Li S, Liu Z, Tian H, Yin X, Huai P, et al. Intake of fruit juice and incidence of type 2 diabetes: a systematic review and meta-analysis. PLoS One. 2014 3 28;9(3):e93471. 10.1371/journal.pone.0093471 24682091 PMC3969361

[10] Moynihan PJ, Kelly SA. Effect on caries of restricting sugars intake: systematic review to inform WHO guidelines. J Dent Res. 2014 1;93(1):8–18. 10.1177/0022034513508954 24323509 PMC3872848

[11] UK and Ireland prevalence and trends. London: Public Health England; 2013. Available from: https://webarchive.nationalarchives.gov.uk/20170110171021/https://www.noo.org.uk/NOO_about_obesity/adult_obesity/UK_prevalence_and_trends [cited 2015 Feb 7].

[12] Statistics on obesity, physical activity and diet: England 2014 [internet]. London: NHS digital; 2014. Available from: https://digital.nhs.uk/data-and-information/publications/statistical/statistics-on-obesity-physical-activity-and-diet/statistics-on-obesity-physical-activity-and-diet-england-2014 [cited 2017 Jul 1].

[13] Adult obesity and type 2 diabetes. London: Public Health England; 2014. Available from: https://www.gov.uk/government/uploads/system/uploads/attachment_data/file/338934/Adult_obesity_and_type_2_diabetes_.pdf [cited 2017 Jul 2].

[14] National Diabetes Audit. London: The Health and Social Care Information Centre; 2008. Available from: https://digital.nhs.uk/data-and-information/publications/statistical/national-diabetes-audit/national-diabetes-audit-2008-09#related-links [cited 2018 Jun 6].

[15] Sugar and health. Postnote: 493. London: The Parliamentary Office of Science and Technology; 2015. Available from: http://researchbriefings.parliament.uk/ResearchBriefing/Summary/POST-PN-0493 [cited 2016 Dec 2].

[16] National Dental Epidemiology Programme for England: oral health survey of five-year-old children 2012. A report on the prevalence and severity of dental decay. London: Public Health England; 2012. Available from: https://webarchive.nationalarchives.gov.uk/20160603145410/http://www.nwph.net/dentalhealth/Oral%20Health%205yr%20old%20children%202012%20final%20report%20gateway%20approved.pdf [cited 2017 Feb 2].

[17] Dental public health epidemiology programme. Oral health survey of three-year-old children 2013. A report on the prevalence and severity of dental decay. London: Public Health England; 2014. Available from: https://webarchive.nationalarchives.gov.uk/20160309201743/http://www.nwph.net/dentalhealth/reports/DPHEP%20for%20England%20OH%20Survey%203yr%202013%20Report.pdf [cited 2017 Jun 6].

[18] Adult dental health survey. Executive summary. London: The Health and Social Care Information Centre; 2009. Available from: https://files.digital.nhs.uk/publicationimport/pub01xxx/pub01086/adul-dent-heal-surv-summ-them-exec-2009-rep2.pdf [cited 2017 Nov 2].

[19] National diet and nutrition survey. Results from years 7 and 8 (combined) of the rolling programme (2014/2015 to 2015/2016). London: Public Health England; 2018. Available from: https://www.gov.uk/government/statistics/ndns-results-from-years-7-and-8-combined [cited 2018 Jun 6].

[20] National diet and nutrition survey. Results from years 5 and 6 (combined) of the Rolling Programme (2012/2013–2013/2014). London: Public Health England; 2016. Available from: https://www.gov.uk/government/statistics/ndns-results-from-years-5-and-6-combined [cited 2017 Jun 2].

[21] Making it happen. Annual report 2017. London: British Soft Drinks Association; 2017. Available from: http://www.britishsoftdrinks.com/write/MediaUploads/Publications/BSDA_Drinks_Report_2017.pdf (cited 2019 Feb 6].

[22] Leading the way. Annual report 2016. London: British Soft Drinks Association; 2016. Available from: http://www.britishsoftdrinks.com/write/MediaUploads/Publications/BSDA_Annual_report_2016.pdf [cited 2018 Jun 6].

[23] Soft Drinks Industry Levy. Policy paper. London: HM Revenue & Customs; 2016. Available from: https://www.gov.uk/government/publications/soft-drinks-industry-levy/soft-drinks-industry-levy [cited 2018 Jun 6].

[24] van Raaij J, Hendriksen M, Verhagen H. Potential for improvement of population diet through reformulation of commonly eaten foods. Public Health Nutr. 2009 3;12(3):325–30. 10.1017/S1368980008003376PMID:18671891 18671891

[25] Hashem KM, He FJ, Jenner KH, MacGregor GA. Cross-sectional survey of the amount of free sugars and calories in carbonated sugar-sweetened beverages on sale in the UK. BMJ Open. 2016 11 15;6(11):e010874. 10.1136/bmjopen-2015-010874 28186923 PMC5128908

[26] Grocery Market Share (12 weeks ending) [internet]. London: Kantar; 2016. Available from: http://www.kantarworldpanel.com/en/grocery-market-share/great-britain [cited 2017 Jun 20].

[27] Dunford EK, Neal B. FoodSwitch and use of crowdsourcing to inform nutrient databases. J Food Compos Anal. 2017;64:13–7. 10.1016/j.jfca.2017.07.022

[28] Guide to creating a front of pack (FoP) nutrition label for pre-packed products sold through retail outlets. London: Department of Health; 2016. Available from: https://assets.publishing.service.gov.uk/government/uploads/system/uploads/attachment_data/file/566251/FoP_Nutrition_labelling_UK_guidance.pdf [cited 2017 Jul 1].

[29] Tesco first to take action on sugar. Action on sugar [internet]. London: Queen Mary University of London; 2015. Available from: http://www.actiononsugar.org/news-centre/press-releases/2015/items/tesco-first-to-take-action-on-sugar.html [cited 2017 Nov 30].

[30] Tesco praised for cutting sugar in own brand drinks [internet]. London: ITV; 2016. Available from: http://www.itv.com/news/2016-11-07/tesco-praised-for-cutting-sugar-in-own-brand-drinks/ [cited 2017 Jun 6].

[31] Dean J. Why we're committed to offering healthier eating options [internet]. Leeds: ASDA; 2018. Available from: https://corporate.asda.com/blog/2018/04/03/why-were-committed-to-offering-healthier-eating-options [cited 2019 Jul 22].

[32] Hipwell D. Sugar in Lucozade reduced by 50% to escape tax penalty. The Times. 2016 Nov 16. Available from: http://www.thetimes.co.uk/article/sugar-in-lucozade-reduced-by-50-to-escape-tax-penalty-996lh697q [cited 2018 Jan 1].

[33] Making good food even better [internet]. Bracknell: Waitrose; 2019. Available from: https://www.waitrose.com/home/inspiration/about_waitrose/about_our_food/sugar-levy-waitrose.html [cited 2019 Jul 23].

[34] Sugar reduction progress update. London: Sainsbury's; 2018. Available from: https://www.about.sainsburys.co.uk/~/media/Files/S/Sainsburys/Sainsburys%20Sugar%20Reduction%20Progress%20Update%202018.pdf [cited 2019 Jul 23].

[35] Irn Bru recipe to change to reduce its sugar content [internet]. Edinburgh: The Royal Environmental Health Institute of Scotland: 2019. Available from: https://www.rehis.com/story/irn-bru-recipe-change-reduce-its-sugar-content [cited 2019 Jul 22].

[36] Sugar reduction and wider reformulation programme: Report on progress towards the first 5% reduction and next steps. London: Public Health England; 2018. Available from: https://assets.publishing.service.gov.uk/government/uploads/system/uploads/attachment_data/file/709008/Sugar_reduction_progress_report.pdf [cited 2018 Jun 8].

[37] Briggs ADM, Mytton OT, Kehlbacher A, Tiffin R, Elhussein A, Rayner M, et al. Health impact assessment of the UK soft drinks industry levy: a comparative risk assessment modelling study. Lancet Public Health. 2016 12 16;2(1):e15–22. 10.1016/S2468-2667(16)30037-8 28804786 PMC5543265

[38] Nourishing framework use economic tools to address food affordability and purchase incentives. London: World Cancer Research Fund; 2018. Available from: London: Error! Hyperlink reference not valid.https://www.wcrf.org/sites/default/files/Use-economic-tools.pdf [cited 2018 Jun 6].

[39] Colchero MA, Popkin BM, Rivera JA, Ng SW. Beverage purchases from stores in Mexico under the excise tax on sugar sweetened beverages: observational study. BMJ. 2016 1 6;352:h6704. 10.1136/bmj.h6704 26738745 PMC4986313

[40] Rogers PJ, Hogenkamp PS, de Graaf C, Higgs S, Lluch A, Ness AR, et al. Does low-energy sweetener consumption affect energy intake and body weight? A systematic review, including meta-analyses, of the evidence from human and animal studies. Int J Obes. 2016 3;40(3):381–94. 10.1038/ijo.2015.177 26365102 PMC4786736

[41] Yang Q. Gain weight by “going diet?” Artificial sweeteners and the neurobiology of sugar cravings: Neuroscience 2010. Yale J Biol Med. 2010 6;83(2):101–8. 20589192 PMC2892765

